# The Deadly Duo of COVID-19 and Cancer!

**DOI:** 10.3389/fmolb.2021.643004

**Published:** 2021-04-12

**Authors:** Vivek R. Bora, Bhoomika M. Patel

**Affiliations:** Department of Pharmacology, Institute of Pharmacy, Nirma University, Ahmedabad, India

**Keywords:** COVID-19, cancer, immunity, drug repurposing, inflammation, anti-cancer drugs

## Abstract

As of September 19, 2020, about 30 million people have been infected with the novel corona virus disease 2019 (COVID-19) globally, and the numbers are increasing at an alarming rate. The disease has a tremendous impact on every aspect of life, but one of the biggest, related to human health and medical sciences, is its effect on cancer. Nearly 2% of the total COVID-19 patients prior to May 2020 had cancer, and the statistics are quite frightening as the patient can be referred to as “doubly unfortunate” to suffer from cancer with the added misery of infection with COVID-19. Data regarding the present situation are scarce, so this review will focus on the deadly duo of COVID-19 and cancer. The focus is on molecular links between COVID-19 and cancer as inflammation, immunity, and the role of angiotensin converting enzyme 2 (ACE2). Complications may arise or severity may increase in cancer patients due to restrictions imposed by respective authorities as an effort to control COVID-19. The impact may vary from patient to patient and factors may include a delay in diagnosis, difficulty managing both cancer therapy and COVID-19 at same time, troubles in routine monitoring of cancer patients, and delays in urgent surgical procedures and patient care. The effect of anti-cancer agents on the condition of cancer patients suffering from COVID-19 and whether these anti-cancer agents can be repurposed for effective COVID-19 treatment are discussed. The review will be helpful in the management of deadly duo of COVID-19 and cancer.

## Introduction

Corona virus disease 2019 (COVID-19) was declared as a pandemic by the World Health Organization by March 2020 when it had affected about 4 million people worldwide and had caused deaths of about 16000 people approximately in 195 countries worldwide (Tay et al., 2020; World Health Organization, 2020 – WHO Director-General’s opening remarks at the media briefing on COVID-19 – March 11, 2020). The pandemic has affected nearly 105 million people as of February 8, 2021, and the cases are still increasing at an alarming rate globally (World Health Organization., 2021 – Coronavirus disease [COVID-19] pandemic 2021). The main reason for the outbreak is the ability of the virus to transmit from one individual to other through air. There is maximum possibility of transmission from one person to another or from one person to 1000 individuals or more, i.e., super-spreading by a super-spreader which makes this COVID-19 extremely deadly (Cave, 2020). Adversity created by the COVID-19 pandemic is humongous and affects each and every aspect of life. The complexity of the COVID-19 pandemic is increasing and the main reason behind this is the current state of people worldwide, i.e., lack of specific immunity to fight against SARS-CoV-2 (Tian et al., 2020).

The disease is of viral origin and the causative agent is the deadly virus, severe acute respiratory syndrome coronavirus 2 (SARS-CoV-2). This virus belongs to a family of coronaviruses which has been affecting humans as well as animals. Coronaviruses in humans infect the upper respiratory tract as well as the lower respiratory tract. The viruses affecting the lower respiratory tract are severe acute respiratory syndrome coronavirus (SARS-CoV), Middle East respiratory syndrome coronavirus (MERS-CoV), and the SARS-CoV-2. The SARS-CoV-2 causing the pandemic in humans has 79% similar genes as in bats and pangolins. The SARS-CoV-2 being a betacoronavirus genus has the ability to cause pneumonia in humans by multiplying in the lower respiratory tract (Peng et al., 2020). This infection to the respiratory system and pneumonia may be life threatening to the individual if not diagnosed and treated in time (Peng et al., 2020; Tay et al., 2020). The risk of infection is high in cancer patients, because cancer patients have decreased immunity against SARS-CoV-2 (Tian et al., 2020).

Cancer is a disease or a group of diseases which is characterized by an abnormal growth of cells which often lead to death. The reason for being prone to cancer can be both genetic and due to lifestyle. Almost every organ of the body is prone to cancer growth; however, there is difference between occurrences of organ specific cancers. As per Globocan 2018, around 18.1 million people were suffering from cancer and 9.5 million people died due to cancer (All Cancers, 2020; NIH National Cancer Institute, 2020). Likewise, whether SARS-CoV-2 can cause cancer is not known yet, but cancer patients and cancer survivors are reported to have two to three times more chances of severe events, suffering, and death along with increased risk to get infected with COVID-19 (Tian et al., 2020). So it is the need of the hour to study the various aspects of “doubly unfortunate” patient suffering from both COVID-19 and cancer. The present review discuses briefly the risk and molecular aspects of the link between cancer and COVID-19, impact on the diagnosis, treatment, and research along with special attention to the repurposing of anticancer drugs in the management of the deadly duo of COVID-19 and cancer.

## COVID-19 in Cancer Patients

### Prevalence

According to the National Cancer Institute, viruses and bacteria have potential roles in the development of cancer. Infections by virus or bacteria are the cause of nearly 1 in 4 case of cancer in developing nations, and nearly 1 in 10 cases of cancer in developed nations (National Cancer Institute (2020) Cancer Prevention Overview (PDQ^®^)–Patient Version).

Respiratory viruses pose more risk to complications in cancer patients. Previously, in 2012 cancer patients had higher death rate after infection with MERS-CoV. In 2015, during the MERS epidemic, in cancer patients the mortality rate was as high as 84% when compared to patients without cancer (Jazieh et al., 2020). Accordingly, the severity of COVID-19 is 76% higher in cancer patients as compared to non-cancer patients (Ofori-Asenso et al., 2020). One study showed that nearly 2% of the total COVID-19 patients prior to May 2020 had cancer, and these statistics are very frightening (Desai et al., 2020). Along with cancer patients being at high risk for COVID-19, there is an increase in the risk of severe conditions, i.e., use of intensive care unit (ICU) and invasive ventilation when compared to non-cancer COVID-19. Data regarding the risk of malignancies and progression are not available yet, nor are data regarding the survival chances of cancer patients with COVID-19 (Tian et al., 2020).

### Risk of Severity

Prognosis or signs and symptoms of COVID-19 in cancer patients are increasing at an alarming rate when compared with non-cancer patients, the difference is of major risk factors that include immunopathology, age, stage of cancer, and inflammation (Xia et al., 2020). The possible cause of this high risk to cancer patients with COVID-19 is surely the immunocompromised state of the patient. 0.79% or 12 patients tested positive for COVID-19 out of 1524 cancer patients admitted in the Department of Radiotherapy and Medical Oncology, Zhonghan Hospital of Wuhan University. At the time of diagnosis, from a total of 1524 cancer patients, 41.7% of patients were undergoing chemotherapy or radiotherapy and immunosuppression was cited as a cause for increased risk of COVID-19 by the authors (Yu et al., 2020). A study conducted in France at the Gustave Roussy Cancer Centre from March 14 to April 15, 2020 reported that the COVID-19 infection rate in cancer patients was 2.1% when compared to the national rate of 0.25% (Barlesi et al., 2020). As a part of their anti-cancer therapy, cancer patients are advised about the use of immunosuppressive agents as well as immune stimulatory agents respective to the cancer patient. The use of these immunosuppressive and immune stimulatory agents leads to an immunosuppressive state of the body in cancer patients (Kalathil and Thanavala, 2016; Tian et al., 2020).

The risk of morbidity was found to be higher in cancer patients after infection with COVID-19; 39%, or seven cancer patients out of 18, suffered from conditions such as admission to the intensive care units or leading to death 8% or 124 non-cancer patients out of 1572 non-cancer patients experiencing intensive care unit admissions or death during the same time. Also, the risk was at 75% in cancer patients who had undergone surgery in the last 30 days and 43% in cancer patients who had not undergone surgery in the last 30 days. The authors explained the reasons of age, smoking, and other existing conditions as risk factors for COVID-19 related risk (Liang et al., 2020). Hematological patients in China and cancer patients in Northern Italy demonstrated similar risks in cancer and COVID-19 (He et al., 2020; Stroppa et al., 2020). A total of 128 hospitalized cancer patients in Wuhan had more susceptibility to COVID-19 and 10% of hematological cancer patients contracted COVID-19. These patients experienced more life-threatening complications as compared to non-cancerous COVID-19 patients (He et al., 2020). In Northern Italy, the study conducted on 25 patients with cancer and COVID-19 showed a high mortality rate of about 36% compared to 16.13% in non-cancerous COVID-19 patients. Lung cancer was prominent among cancer patients and more severe conditions like shortness of breath, bilateral interstitial abnormalities (ground-glass opacities), shadowing, and crazy paving observed in chest x-ray and chest CT scan were experienced by lung cancer patients, the factors of risk were justified as age, sex (females were at higher risk than males), and the antiviral therapy used for management of COVID-19. Interestingly, patients receiving pembrolizumab as immunotherapy did not experience life-threatening conditions (Stroppa et al., 2020). A study showed that the risk of severe outcomes like acute respiratory distress syndrome (ARDS), admissions to ICU, pulmonary embolism, and septic shock was higher in cancer patients with COVID-19 infection, the study had 536 non-cancer patients and 105 cancer patients and interestingly the frequency of severe outcomes was relatively high in cancer patients with hematological cancers, lung cancers or stage IV metastatic cancers (Dai et al., 2020).

The surge in innate immunity cells, mainly cytokines which cause inflammation and infiltration of neutrophils and lymphocytes, which is also termed as a ‘cytokine storm,’ is responsible for the severe conditions experienced by cancer patients due to COVID-19 infection. This cytokine storm also causes bronchoalveolar fluid accumulation in the lungs and causes injury to the lungs. This results in the reduced functional capacity of the lungs. Thus cytokine storm is also a reason for more fatalities in the deadly duo of COVID-19 and cancer. In both cancer and COVID-19, inflammation is experienced by patients. The inflammation is systemic and thus further increases the suffering of cancer patients (Tian et al., 2020).

Hematological cancer, lung cancer, and breast cancer patients have more vulnerability toward getting infected with Sars-CoV-2 (Derosa et al., 2020; Rugge et al., 2020). The incidence of mortality in lung cancer patients infected with SARS-CoV-2 was reported to be up by four times. Breast and hematological cancer patients had to face more hospitalization and death (Rugge et al., 2020). In thoracic cancer patients with COVID-19, a history of smoking, associated comorbidities, and ongoing chemotherapy were all recognized for increased death risk (Garassino et al., 2020). Pediatric cancer patients had less susceptibility toward infection with SARS-CoV-2, contrasting to 14.7% infection rate in asymptomatic caregivers who suffered from COVID-19, only 2.5% of pediatric cancer patients suffered from COVID-19 (Albiges et al., 2020; Boulad et al., 2020).

In some cancers, the patient’s age can be an important risk factor which makes the cancer patients prone to COVID-19 infection. The age factor decreases the cancer patient’s potential to acquire natural immunity against the COVID-19 and worsens the suffering of the cancer patient. The stage of cancer is also an important risk factor in cancer patients, and some patients may be going through radiotherapy or some cancer patients may have undergone surgical procedures (Tian et al., 2020; Xia et al., 2020).

## Molecular Link Between Cancer and COVID-19

Chronology to be understood in COVID-19 is that the virus enters through the respiratory tract in the human body and enters the lungs. The virus enters through air droplets and binds with the specific cells called Type 2 pneumocytes having angiotensin-converting enzyme 2 (ACE2) and transmembrane protease serine 2 (TMPRSS2) (Peng et al., 2020). Spike proteins present on the virus help in binding with the ACE2. After binding multiplication of the viral DNA takes place, thus there is an increase in the viral load in the body. The virus exits the cells through exocytosis, enters the blood stream, and spreads within the body. Simultaneously the host or body reacts to the entry of the virus by increasing the secretion of pro-inflammatory cytokines and chemokines, along with the rise in macrophage and alveolar secretion and initiation of pyroptosis (M. Yang, 2020). At this stage, symptoms such as fever and headache are experienced by the host. Thus, huge macrophage infiltration and cytokine infiltration affects the lung’s capacity to perform its function, and the alveolar capacity is reduced due to the accumulation of bronchoalveolar lavage fluid. The symptoms experienced at this stage are difficulty in breathing, hypoxemia, and pneumonia. Further, due to the systemic spread of the virus, the risk of secondary infection is increased, which can be to the central nervous system, renal system, digestive system, and cardiac system. Terminally acute respiratory distress syndrome (ARDS) is experienced by the host which may be accompanied by multiple organ failure (Tay et al., 2020).

The series of events taking place in COVID-19 helps us understand the molecular mechanism of COVID-19. The coronavirus is a positive sense single-stranded RNA genome with a molecular weight ranging from 26-32 kb. Of the previously identified α, β, γ, and δ genera of coronaviruses, the novel SARS-CoV-2 belongs to β-CoV strain (Li et al., 2020). There are 10 open reading frames (ORFs) in SARS-CoV-2, and viral RNA is translated into large polyproteins; these large polyproteins comprise two-thirds of the ORFs. In viral RNA, these large proteins are present in the rough endoplasmic reticulum, and these are the places where both the transcription and replication of the virus occur. The remaining one-third of ORFs converts into four main structural proteins, spike (S), envelope (E), nucleocapsid (N), and the membrane proteins (M) of the virus (Li et al., 2020). The spike proteins (S) are responsible for the binding and entry of the virus in the host. As discussed above, after the entry of the virus into the host or human, three mechanisms occur to bind with the angiotensin-converting enzyme 2 (ACE2) and transmembrane protease serine 2 (TMPRSS2) receptors and further trigger in the replication of the virus in the host, followed by the initiation of a cytokine storm and hyperactivation of coagulopathy. The virus binds independently with ACE2 and TMPRSS2, the ACE2 binding facilitates the uptake of virus by host cells and the TMPRSS2-binding-activated glycoproteins facilitate the entry of virus into host cells (Li et al., 2020; van Dam et al., 2020).

**FIGURE 1:**
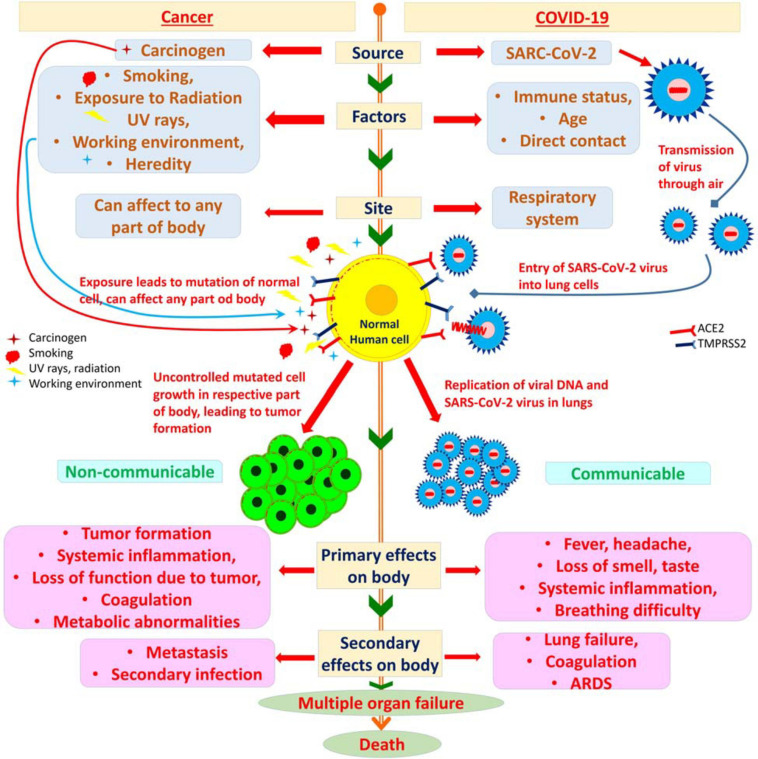
Link between cancer and COVID-19. On the left, cancer progression is illustrated. Cancer is a disease caused by exposure to carcinogens, factors such as smoking, exposure to radiation, UV rays, working environment, and heredity can increase the risk of cancer. Any part of the body can be mutated resulting in cancer. After mutation there is uncontrolled growth of the mutated cells leading to the formation of a tumor, the tumor is non-communicable. Systemic inflammation, loss of function of the respective organ or part of body, coagulation, and metabolic abnormalities are the primary effects of cancer. As cancer progresses to advanced stages, metastasis along with increased susceptibility to secondary infections is observed leading to multiple organ failure and ultimately death. On the right, COVID-19 progression is illustrated. The source of COVID-19, a viral infection, is SARS-CoV-2, risk factors include immune status, age, and direct contact with the virus or infected person. The virus enters through the respiratory system into the human body and gets entry into the lung cells, where the replication of the virus occurs. COVID-19 is highly communicable and leads to fever, headache, loss of smell and taste, along with systemic inflammation and difficulty in breathing. Secondary infection to other organs is observed if the infection is chronic leading to lung failure, coagulation, and acute respiratory distress syndrome. Multiple organ failure leading ultimately to death is observed in COVID-19.

Interestingly, the series of above molecular events also occurs in cancer patients, such as a cytokine storm is observed in cancer, ACE2 and TMPRSS2 expression is found higher in cancer patients, and coagulopathy is a potential risk observed in a number of cancer patients. Thus, these events establish a molecular link between the deadly duo of COVID-19 and cancer (Figure 1).

### Cytokines

Immunosuppressive cytokines are increased in cancer, and these are responsible for the immunity state of the body. There is a gradual increase in cytokines after COVID-19 infection. In asymptomatic patients, cytokine-mediated immunity combatsCOVID-19 before infection enters in advanced stage and the virus is neutralized. In some patients, viral infection by SARS-CoV-2 can trigger a cytokine storm in many symptomatic patients and leads to hyper inflammation or hyperinflammatory syndrome, along with hypercytokinemia, multi-organ failure, and death in some cases. In the lungs, the cytokine storm leads to bronchoalveolar fluid accumulation, inflammatory cell infiltration, squamous metaplasia of epithelial cells and intra-alveolar hemorrhage, vascular congestion especially in type 2 pneumocytes, and multinucleated syncytial cells (Ritchie and Singanayagam, 2020; van Dam et al., 2020).

The cytokine storm is the main cause of acute respiratory distress syndrome (ARDS) and death due to COVID-19. Neutrophilia, i.e., excessive accumulation of mucus in the lungs, is responsible for decreased functioning and capacity of the lungs. Lung dysfunction, pulmonary fibrosis due to surge in epithelial cell proliferation, and tissue remodeling impairment are observed due to infiltration of pro-inflammatory cytokines. Pro-inflammatory cytokines and chemokines are increased in COVID-19, one of the reasons being the activation of the NF-κB and STAT3 pathways, leading to the activation of IL-6, which results in the release of pro-inflammatory cytokines and chemokines, activated T-cells, and activated B-cells. Neutrophil extracellular traps (NETs) are observed in excess due to neutrophilia and lead to thrombosis in arteries and veins. This state is observed in COVID-19 patients with severe infection (Thålin et al., 2019; van Dam et al., 2020).

Inflammation drives the rate of cancer progression and metastasis. Stage and type of cancer are important factors to consider, and the rate at which the cytokine storm occurs after COVID-19 infection defines the severity of infection, suffering, and treatment strategy for the deadly duo of cancer and COVID-19. Cytokine storms observed due to the release of excessive quantity of IL-6, IL-12, IL-18, TNF-α, CCl2, and other cytokines and chemokines prove to be major contributors for ARDS (Huang et al., 2020; Li et al., 2020). The host responds to the brutal strike exhibited by the cytokine storm in the form of hyperactive immune response and thus ARDS. The condition can lead to the failure of multiple organs or in extreme cases lead to fatality in cancer patients with COVID-19 infection (Li et al., 2020).

Clinical investigation of IL-6 and IL-10 can be helpful in predicting the susceptibility of COVID-19 in cancer patients (Ritchie and Singanayagam, 2020; van Dam et al., 2020). Based on the above data the chemokines and cytokines can serve as diagnostic markers for the estimation of the severity of the condition and management of the deadly duo of COVID-19 and cancer.

### Angiotensin Converting Enzyme 2 (ACE2)

The SARS-CoV-2 gains entry into the human body via interaction with ACE2. As discussed earlier, the pathogenesis starts from the binding of SARS-CoV-2 with ACE2 and continues to have an impact on symptoms during the course of the disease. ACE2 as such plays role in the maintenance of normal blood pressure by the renin-angiotensin-aldosterone system (RAAS) (van Dam et al., 2020). Lung and heart cells have higher expression of ACE2 receptors, which facilitates the quick binding of SARS-CoV-2 entering from the respiratory system and ACE2 levels increase. Thus, ACE2 is associated with hypertension and diabetes. In a secondary infection after the lungs, SARS-CoV-2 binds to the ACE2 receptor present on kidneys, liver, bladder, stomach, and intestine. The presence of ACE2 throughout the body makes it more susceptible to cardiac-related ailments, diabetes, and infection to other body organs. All of these are the probable reason for multi-organ failure and even death (Tian et al., 2020; Zhou et al., 2020).

Angiotensin converting enzyme 2 plays an important role in the development of cancer. Inflammation, accumulation of cytokines, vasoconstriction, and increased permeability by vascular endothelium are common, both in cancer and in COVID-19 patients (Ferrario et al., 2005; van Dam et al., 2020). The expression of ACE2 is higher in some cancers such as lung, cervical, pancreatic, and renal carcinomas, while the expression is decreased in breast, prostate, and liver cancers. The condition depends mainly on the type of tumor and stage of cancer. RAAS has a crucial role in cancer, and it is responsible for cell proliferation, angiogenesis, inflammation due to cancer, immunomodulation due to cancer, upregulation of cytokines, growth factors, and transcription factors. Cumulatively, all of these conditions lead to suppressed immunity in cancer patients. Increased expression of ACE2 not only indicates more chances of COVID-19 infection, but also increases in neutrophils and cytokines, macrophage infiltration, and dendritic cell infiltration. This causes increased fluid accumulation as an accumulation of bronchoalveolar fluid in lungs (van Dam et al., 2020).

The consumption of tobacco is a prognosis factor for COVID-19 in cancer patients and this may be due to the fact that many cancer patients have or may have had smoking-related habits. The link between smoking can be established to COVID-19 and cancer as the gene expression of ACE2 is higher with tobacco use (Stroppa et al., 2020; Xia et al., 2020). So, increased expression of ACE2 means increased binding with SARS-CoV-2 (Xia et al., 2020).

Angiotensin converting enzyme 2 is expressed in many organs of the respiratory, digestive, cardiovascular, and urinary systems (Jyotsana and King, 2020; Tian et al., 2020). The ACE2 levels in healthy humans and in those suffering from cancer, COVID-19, or both are different. This difference can be related to the varying expression of ACE2 in different organs of the body, and it is related to different cancer types (Jyotsana and King, 2020). Sadly, up to now, data showing expression of ACE2 with respect to patients with specific types of cancers and COVID-19 are not available to firmly establish a mechanistic link and more studies are required.

### Transmembrane Protease Serine 2 (TMPRSS2)

Host immunity is mediated by a family of proteases called type II transmembrane serine proteases (TTSPs). One member of the family is TMPRSS2. TMPRSS2 is a binding site for SARS-CoV-2, and the bonding takes place with the formation of S protein cleavage at S1/S2 and the S2’ site. Thus, the immune state of the patient is directly proportional to the severity of infection in patients suffering from COVID-19 (Tay et al., 2020; van Dam et al., 2020). The transcription of TMPRSS2 in the lungs is altered by the modulation of androgen and androgen derivatives causing a reduction in androgen levels in prostate cancer patients, which directly affects the chances of COVID-19 infection and severity in patients (Montopoli et al., 2020). Patients, particularly with prostate cancer, have higher expression of TMPRSS2 as compared to patients with renal, lung, colorectal, or pancreatic cancers, while other cancers have no significant expression of TMPRSS2. So, the risk of lung cancer patients having TMPRSS2 expression is moderate but it is higher in prostate cancer patients (van Dam et al., 2020).

### Coagulation

Imbalance in the normal levels of fibrinogen, anti-thrombin III, and D-Dimers leading to coagulopathy is observed in COVID-19 patients (Wang et al., 2020). The coagulopathy is mostly prothrombotic and is a result of COVID-19 infection. There is a direct involvement of coagulation factors and platelets, which are altered due to increased cytokines. Pulmonary congestion, arterial occlusive events, venous thromboembolism, and especially lung microvascular thrombosis are observed in COVID-19 patients. These events are persistent in patients where ARDS is reported (Magro et al., 2020); 71.4% of dying patients have reported intravascular coagulation compared to 0.6% in surviving patients (van Dam et al., 2020; Wang et al., 2020).

In cancers, the coagulation risk is a well-known condition arising due to the presence of cancers and risk associated with thrombosis depending upon the stage of cancer, timely diagnosis, and the anti-cancer therapy. Events such as thrombotic microangiopathy and disseminated intravascular coagulation are observed in cancer patients. Tumoral factors such as tissue factor (TF), podoplanin, plasminogen activator factor (PAI-1), cytokines, NET, and mucins trigger the risk for thrombosis (Thålin et al., 2019). The type of cancer changes the risk severity of coagulation, e.g., adenocarcinomas, lung cancer, pancreatic cancer, gastrointestinal cancer, and ovarian cancer have elevated risk for coagulation, while the risk is lower in breast and renal carcinoma compared to no risk associated with prostate cancer and melanoma (van Dam et al., 2020). During a pathological examination of a COVID-19 patient with cancer, it is essential to identify the risk associated with coagulation and the data will be helpful in the management of the deadly duo of COVID-19 and cancer.

## Impact of COVID-19 in Cancer

### Impact on Diagnosis

According to a report by the Netherland Cancer Registry, there was a reduction in the diagnosis of cancer by 26% excluding skin cancer, while skin cancer diagnosis was reduced by 60% between February 24, 2020, and April 12, 2020 (Dinmohamed et al., 2020). A 75% drop in referral cases for an early diagnosis of cancer was observed and loss of approximately 18000 lives was estimated in the United Kingdom alone due to the reduction in the rate of diagnosis of cancer patients. The United Kingdom is among the top countries for cancer care throughout the world, and its health system was near to failure in tackling COVID-19 (Sud et al., 2020).

Such drastic impact has been experienced all over the world in regards to health systems and especially cancer care. Diagnosis is a very essential first step in cancer care. Delays in diagnosis may have severe effects on the patient as it delays everything from managing symptoms, to curing the cancer, and monitoring as well. Many countries have been advised to delay cancer care due to the COVID-19 pandemic as a measure to curb the transmission of COVID-19, thus degrading the quality of life in cancer patients. Also, the risk for health care providers and family members is increased due to frequent visits to hospitals which are essential, and when community transmission of COVID-19 takes place, making the decision to delay cancer diagnosis unavoidable.

**FIGURE 2:**
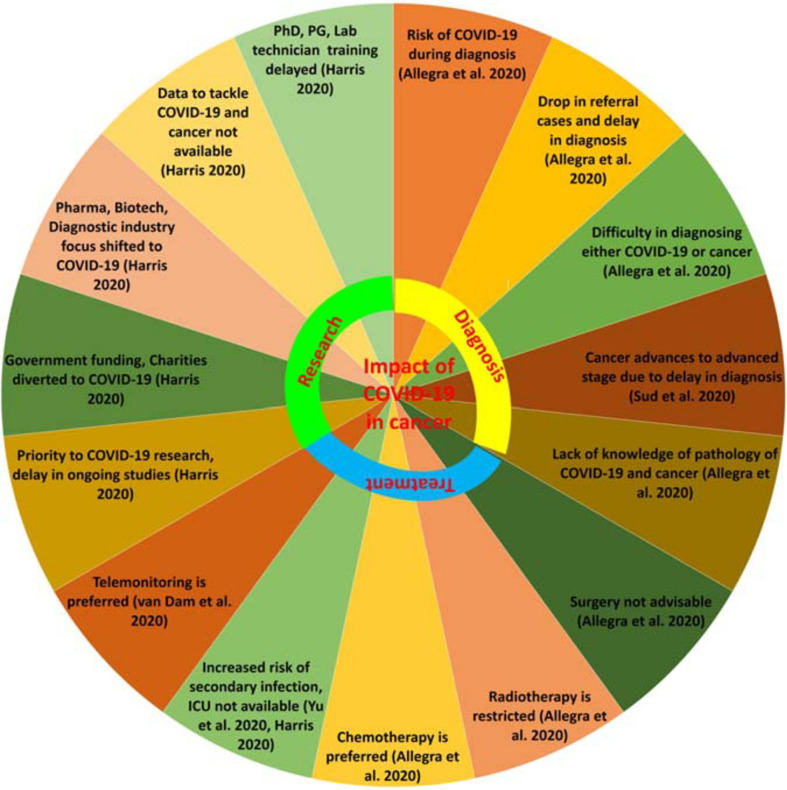
Impact of COVID-19 on cancer. Impact on diagnosis, treatment, and research.

The presence of the deadly duo of COVID-19 and cancer makes diagnosis very difficult. Diagnosis of radiographs can be similar in both COVID-19 and cancer which may deceive the healthcare professional in making an accurate diagnosis. Common markers in both COVID-19 and cancer are carbohydrate antigens (CA) 125 and 153, carcinoembryonic antigens (CEA), human epididymis protein 4 (HE4), C-reactive protein (CRP), and cytokeratin-19 fragment (CYFRA21-1); these markers are increased in both COVID-19 and cancer. But, to diagnose whether the rise in these markers is due to COVID-19 or cancer or both at the same time poses a challenge for the healthcare professional (Allegra et al., 2020). Impact on diagnosis is crucial and requires deep knowledge of the pathologies of the deadly duo of COVID-19 and cancer (Allegra et al., 2020) (Figure 2).

### Impact on Treatment

As cancer care is affected, treatment is also affected. The delay in diagnosis completely changes the parameters of cancer treatment. The treatment is to be done while keeping the additional risk factors of COVID-19 associated with cancer. Certain anti-cancer therapy may require a compromise of immunity in order to kill cancer cells. This state of the patient makes them more susceptible to acquiring the SARS-CoV-2 infection. Proper planning is needed in the management of radiotherapy and having small sessions instead of a single session is not preferable. Additionally, it is suggested to reduce the frequency of the small sessions, and radiotherapy sessions must be properly set so as to restrict the chances of COVID-19 infection during radiotherapy. Chemotherapy wherein hospitalization is not required should be preferred. Oral anticancer therapy can help to address this condition.

There is evidence that anti-viral therapy alters the anti-cancer treatment. Delaying the chemotherapy in hepatitis C virus (HCV)-positive cancer patients is advised while antiviral therapy is in progress (Borchardt and Torres, 2014). Post-vaccination for influenza in patients suffering from ovarian cancer it was observed that patients were not able to generate an antibody response during the course of chemotherapy (Chu et al., 2013). Liver damage due to infection by hepatitis B virus is observed in 20% of cancer patients undergoing chemotherapy (Yeo et al., 2000). Reactivation of tuberculosis is reported in cancer patients after chemotherapy (Jacobs et al., 2015).

Similarly, the COVID-19 treatment and cancer treatment may interfere with each other, but to conclude how chemotherapy interferes with COVID-19 treatment or vice versa, the data is not available. The possibility of re-infection with SARS-CoV-2 exists and can be a risk for anti-cancer treatment in cancer patients after recovery from COVID-19, the use of immunosuppressive agents as anti-cancer therapy can be a risk factor for COVID-19 re-infection (Bi et al., 2020). To avoid more suffering to patients from the deadly duo of COVID-19 and cancer, oncologists along with cancer societies advise putting cytotoxic chemotherapy on hold and waiting until the SARS-CoV-2 virus becomes negative in the body (Al-Shamsi et al., 2020; Bi et al., 2020).

In actual practice, postponing the cancer treatment has its own consequences ranging from progression of the cancer state to arising of other complications like anxiety. To decide whether to initiate chemotherapy and when to initiate chemotherapy after recovery from COVID-19 is dependent on the condition of the cancer patient as using immunosuppressive anti-cancer agents can reactivate the SARS-CoV-2 virus leading to the reactivation of COVID-19 (Bi et al., 2020). Cancer patients receiving chemotherapy are more prone to pneumonitis, neutropenia like severe conditions in comparison to cancer patients not receiving chemotherapy (Liang et al., 2020). The multiple organ failure rate with respect to COVID-19 is high, specifically liver dysfunction (29%), acute kidney injury (29%), and cardiac injury (23%), but whether chemotherapy has its impact on multiple organ failure or dysfunction in cancer patients is not known as of yet (Bi et al., 2020; Yang et al., 2020).

The study outcomes of Bi et al. (2020) revealed that, from a total of 39 cancer patients in the study, one adverse event of grade I or II associated with therapy was observed in 31 (79%) of patients. Among these, four cancer patients experienced neutropenia of grade III or IV. The condition eased in respective cancer patients after treatment with granulocyte colony-stimulating factor (G-CSF). Interestingly, the cancer patients included in the study had recovered from COVID-19 and were negative to SARS-CoV-2 before the initiation of chemotherapy, and these cancer patients had at least one anti-SARS-CoV-2 antibody present in their body (Bi et al., 2020).

There is the possibility of the patient not having COVID-19 at the time of surgery or radio therapy. But, due to the immunocompromised state of patient during anti-cancer therapy, chances to acquire COVID-19 are increased. Such cases were reported, and 34 subjects developed pneumonia after surgery. The reason behind the pneumonia was attributed to SARS-CoV-2 acquired after the surgery, 44% of patients had to be kept in ICU and half of these died. Concurrently, the progression of cancer can be a challenge of delaying surgeries in cancer patients respective of the stage of cancer. Delays in the case of prostate, breast, cervical, or skin cancer in early stages can be tolerated but pancreatic, lung, and hematological cancers such as leukemia require treatment as soon as possible (Allegra et al., 2020).

Healthcare professionals need to work with more pressure and follow all the protective measures for the prevention of COVID-19, make use of newer tools such as telemonitoring, and make use of artificial intelligence to increase the timely treatment in cancer patients and also to minimize risk factors associated with anti-cancer treatment (Peeters et al., 2020; van Dam et al., 2020).

**FIGURE 3:**
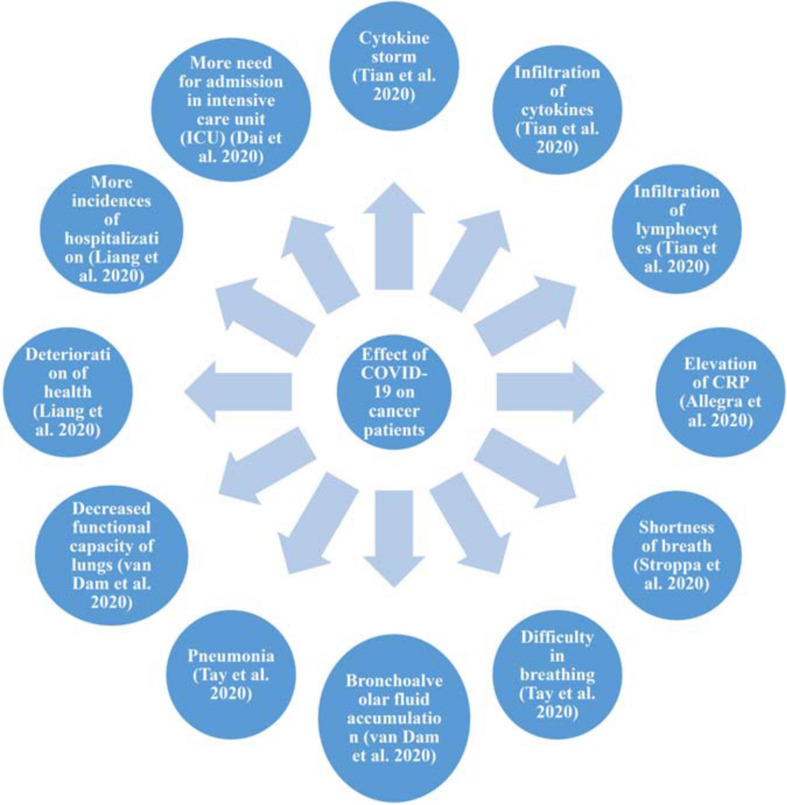
Effect of COVID-19 on cancer patients. Effect of COVID-19 on cancer patients with respect to inflammation, and changes to the respiratory system, health, and quality of life.

The National Health System (NHS), United Kingdom, has issued guidelines for the prioritization of cancer care with priority level from 1 to 6, where factors such as curative and non-curative aspects of treatment, surgery, radiotherapy, adjuvant treatment, success percentage, and survival time are considered (Allegra et al., 2020). These kinds of efforts are required by all countries along with active participation from Oncological societies to help with the treatment of the deadly duo of COVID-19 and cancer (Figures 2, 3).

### Impact on Cancer Research

One major area to reassess during COVID-19 is cancer research. Due to the top most priority being given to COVID-19 management, ongoing trials in the cancer field are on hold. No new trials are to be started in the near future while the pandemic situation continues. Several sensitive techniques such as tumor-infiltrating lymphocytes (TILs) and chimeric antigen receptor (CAR)-T cells have been on hold since the pandemic began as these require intensive care units (ICU) to be available. The complete focus of pharmaceutical companies, the biotechnology industry, and the diagnostics industry is shifted to COVID-19, resulting in a stay of ongoing studies and developments. Universities, which are centers of innovation, are closed, not only stopping the flow of knowledge but also delaying training of Ph.D. students, postdoctoral students, undergraduate, and postgraduate students. Also, the training of laboratory technicians is on hold. Funding and grants to principal investigators are stopped. Funding sources are majorly from charities by people, which are reduced due to the COVID-19 pandemic. Government agencies are the major contributors for financing research which have been focused on the COVID-19 pandemic. The use of data to manage the deadly duo of COVID-19 and cancer is necessary, and thus, evolution in artificial intelligence can be useful in the near future (Harris, 2020) (Figure 2).

## Anticancer Agents in COVID-19

With the ongoing COVID-19 pandemic, it is quite challenging to design a fool-proof therapy for the management of cancer. As concluded by di Lorenzo et al. (2020), while designing therapy for the management of the deadly duo of COVID-19 and cancers, some key points should be considered, including possible interactions that can occur due to treatment measures, the severity of the disease, etc. The use of agents in the management of COVID-19 should not only be efficient in eliminating the SARS-CoV-2 infection but also serve as a dual agent in the treatment of cancer (di Giacomo et al., 2020). If chemotherapy or radiotherapy is started prior to COVID-19 infection, then potential risk and benefits in stopping anticancer treatment should be considered. Agents used for anticancer therapy should be wisely selected and the regimen for the same should be decided appropriately (di Lorenzo et al., 2020).

### Repurposing of Different Anticancer Agents in COVID-19

One or more pharmacological agents may be needed in the management of the deadly duo of cancer and COVID-19, and none of the anti-cancer or anti-viral agents used along with other pharmacological agents should act as obstacles while providing treatment and care to the patients (di Giacomo et al., 2020). Different mechanisms of anti-cancer agents that are being investigated for repurposing in the treatment of COVID-19 are to restrict the replication of the SARS-CoV-2 virus, preventing the infection of host cells by SARS-CoV-2, restricting hyperactivation of the immune system, inhibiting cytokine inflammation and infiltration of immune cells, and preventing lung related pathological conditions (Borcherding et al., 2020). Notably, data related to anti-cancer agents belonging to interleukin (IL) inhibitors, immunomodulators, androgen biosynthesis inhibitors, complement system inhibitors, immune checkpoint inhibitors, bruton tyrosine kinase inhibitors, and Janus-associated kinase (JAK) inhibitors currently under trials for the treatment of COVID-19 are available. Some agents have proved to be beneficial while some have not (Borcherding et al., 2020; El Bairi et al., 2020; Saini et al., 2020). No agent is approved as of now and limited data is available regarding their repurposing in COVID-19 treatment.

#### Interleukin (IL) Inhibitors

Tocilizumab, Sarilumab, Anakinra, and Siltuximab are some of the interleukin inhibitors used in the management of cancer; these drugs are cytokine inhibitors and help in curbing the inflammation, modulating immune responses induced by cancer itself, or curbing inflammation and immune-related side effects of anticancer agents. These interleukin inhibitors are available in injection and pill form and can be administered as an infusion or by the oral route. These agents may have potential in COVID-19 and cancer management, as cytokine storms are common events observed in both the diseases. Tocilizumab, an interleukin-6 (IL-6) inhibitor, is used to counter cytokine release syndrome aroused due to CAR-T anticancer therapy. In CRT therapy, immune cells are used as anticancer agents, and this causes cytokine storm release by the body (di Giacomo et al., 2020; di Lorenzo et al., 2020). Sarilumab and Siltuximab are interleukin-6 (IL-6) inhibitors implied for the treatment of rheumatoid arthritis and Castleman disease, respectively, and can be used in the management of cancer patients with COVID-19. Anakinra, an inhibitor of interleukin-1 (IL-1), can be used in ARDS and can be helpful in controlling cytokine storms in COVID-19 (di Lorenzo et al., 2020). As inflammation and cytokine release is an important factor in both COVID-19 and cancer, these cytokine inhibitors can be repurposed for the treatment of COVID-19.

#### Immunomodulators

Emapalumab is an interferon-γ (IFN-γ) inhibitor used in the treatment of hairy cell leukemia, melanoma, and follicular lymphoma (Saini et al., 2020). It is available in injection form. Interferon-γ (IFN-γ) excessive production is nullified by using Emapalumab in children suffering from hemophagocytic lymphohistiocytosis (HLH) and keeps the children alive until a transplant can be made (di Lorenzo et al., 2020). Immunomodulators such as thalidomide and lenalidomide implied for treatment of multiple myeloma are currently under trial for their efficacy in COVID-19 (Saini et al., 2020).

#### Androgen Biosynthesis Inhibitors

Abiraterone, a hormone used in anticancer treatment in prostate cancer, requires immunosuppression to act effectively, and this condition can increase the risk of COVID-19 infection (de Bono et al., 2011). Abiraterone is available in pill form and can be administered orally. Promising results have been reported in *in vitro* studies as Abiraterone has the potential to obstruct the SARS-CoV-2 viral replication in VeroE6 and Caco2 cells (Yuan et al., 2020).

#### Complement System Inhibitors

Eculizumab is a complement system inhibitor which can be given by intravenous infusion. It targets the complement protein 5 and is used in the treatment of paroxysmal nocturnal hemoglobinuria (PNH) and atypical hemolytic urea syndrome (aHUS). Eculizumab is under clinical trial for repurposing in the treatment of COVID-19 at Hudson Hospital (Tay et al., 2020).

#### Immune Checkpoint Inhibitors (ICI)

Use of immune checkpoint inhibitors (ICI) is associated with an increased risk of pneumonia. Toxicity related to ICI and IL-6 has been decreased with the use of Tocilizumab (Naqash et al., 2018). In patients with the deadly duo of COVID-19 and cancer, no cases of immunotoxicity were reported. Camrelizumab is a potential candidate available in injection form to be administered by infusion which can be used in the management of COVID-19; however, the study is not completed as of now and results are awaited (Dolgin, 2020). Reduction in sepsis or infection after pneumonia and inflammatory response syndrome was observed in COVID-19 patients administered with PD-1 inhibitors. PD-1 inhibitors are ICI, which have gained potential importance in solid cancer treatment (Allegra et al., 2020).

#### Bruton Tyrosine Kinase (BTK) Inhibitor

The potential of ibrutinib is being evaluated. It is available in tablet, capsule, and injection form but oral administration is preferred over infusion as it does not require visits to the hospital. A Bruton tyrosine kinase (BTK) inhibitor and hematopoietic cell kinase (HCK) inhibitor are used in the treatment of chronic lymphocytic leukemia, mantle cell lymphoma, and Waldenstrom’s macroglobulinemia. Ibrutinib is capable of reducing damage to the lungs, and reducing the concentration of pulmonary cytokines causing inflammation and is proven to decrease death in H1N1 influenza virus in animals. However, ibrutinib is associated with the risk of hypoxia, but ibrutinib can be a potential anticancer agent that can be repurposed for COVID-19 treatment (Florence et al., 2018; Allegra et al., 2020; Treon et al., 2020).

#### Janus-Associated Kinase (JAK) Inhibitors

Ruxolitinib and baricitinib are being studied for repurposing in COVID-19 treatment. Ruxolitinib and baricitinib can be given by oral route as it is available in tablet form. These are Janus-associated kinase (JAK) inhibitors used in the treatment of myelofibrosis and polycythemia vera and other hematological malignancies. Ruxolitinib has been reported to reduce the cytokine mediated inflammation, reducing severe events such as ARDS in COVID-19 infected patients, and many trials are currently active (Borcherding et al., 2020).

### Drug Interactions

The drug interactions are very crucial factors to consider while designing anticancer therapy with the risk of COVID-19. Some of the reported interactions report that the incidence of interactions is not high. Some important interactions reported (Table 1) are between chloroquine, hydroxychloroquine, and trastuzumab resulting in ventricular hypertrophy, valvular dysfunction, and conduction disorders (Gautret et al., 2020). Interaction between antiviral drugs and androgen receptor signaling inhibitor (ARSI), Enzalutamide, is predicted, probably due to interference between cytochromes. Notably, Enzalutamide is used in the treatment of prostate cancer (Nhean et al., 2018).

**TABLE 1:** Anti-cancer agent interaction with COVID-19 agent.

**Anti-cancer agent**	**Interaction with anti-COVID-19 agent**	**Mechanism**	**Effect**
Regorafenib	Azithromycin	Multikinase inhibitor specially inhibits tyrosine kinase	Reduction in therapeutic potential
Vinblastin	Azithromycin	Inhibition of mitosis leading to cell death	P-glycoprotein serum levels are increased
Doxorubicin	Chloroquine	Inhibition of topoisomerase II and initiation of apoptosis	Cardiac related abnormalities
Trastuzumab	Chloroquine	Inhibition of HER2	Cardiac related abnormalities
Fluorouracil	Anakinra	Inhibition of thymidylate synthase	Suppression of immunity
Fluorouracil	Tocilizumab	Inhibition of thymidylate synthase	Suppression of immunity
Durvalumab	Anakinra	Inhibition of PD-L1	Reduced therapeutic efficacy
Enzalutamide	Favipiravir	Competitive binding of androgen, inhibition of tumor gene transcription	Reduction in action of antiviral agent
Abiraterone	Tocilizumab	Androgen biosynthesis inhibitor	Tocilizumab reduced abiraterone as CYP3A4 inducer
Abiraterone	Colchicine	Androgen biosynthesis inhibitor	Increase action of abiraterone as CYP3A4 inhibitor
Docetexel	Tocilizumab	Inhibition of microtubular depolymerization	Tocilizumab reduced docetexel as CYP3A4 inducer
Ruxolitinib	Tocilizumab	Inhibits myelofibrosis and JAK	Tocilizumab reduced ruxolitinib as CYP3A4 inducer
Paclitaxel	Tocilizumab	Inhibits cell division by altering chromosomal segregation	Tocilizumab reduced paclitaxel as CYP3A4 inducer

Increased metabolism of anticancer agents such as ceritinib, criotinib, brigatinib, gefitinib, and docetexel is observed, thus decreasing the efficacy of anticancer therapy. These are especially used in lung cancer. Antiviral drug Tocilizumab is known to elevate cytochrome P450 (CYP) and isoenzyme CYP3A4 involved in the metabolism of the above mentioned drugs. Immune checkpoint inhibitors (ICI) (durvalumab, nivolumab, and atezolizumab) activity is decreased as tocilizumab has the potential to suppress the immune response (Ying et al., 2015). ICIs are used in the management of various cancers, such as lung, kidney, gastric cancer, and thyroid cancer. Drug interaction between immune checkpoint inhibitors and anakinra and ruxolitinib is not established but risk factors must be considered before administering respective drugs (di Lorenzo et al., 2020). Cisplatin and vinca alkaloids are potent anticancer agents, and their interaction with colchicine and tocilizumab is not established yet (La Regina et al., 2019).

## Conclusion

Measures to control the spread of COVID-19 are carried out throughout the world. Many countries like United States, Brazil, France, Spain, United Kingdom, Russia, Italy, Mexico, India, and Indonesia, among others, are facing a second wave of COVID-19 as spikes in the number of cases are observed from September 2020 to January 2021 (Johns Hopkins Corona Resource Center, 2021 – New Cases of COVID-19 in World Countries). The current scenario is quite frightening for cancer patients, if patients come in contact with COVID-19 then managing the patient is a challenge for health care systems globally. As the severity of the disease increases, there is an increase in risk for providing symptomatic care to COVID-19 patients suffering from cancer. The impact on cancer diagnosis as well as treatment is worsening for the patient and even become unmanageable, leading to deaths of patients. The molecular mechanism is quite clear between cancers and COVID-19 where ACE2, cytokines, TMPRSS2, and coagulation are prominent. Joint efforts by the healthcare system and oncological societies are very crucial and will be very fruitful for the patient. Some anticancer drugs like cytokine inhibitors, immune checkpoint inhibitors, Bruton tyrosine kinase inhibitors, and hormonal therapy can be repurposed for combating the deadly duo of COVID-19 and cancer. Considering few reports of drug interactions between anticancer and anti-viral treatment, anti-cancer drugs can be potential targets for treating the deadly duo of COVID-19 and cancer. Data available are quite scarce, so to confirm the repurposing of anticancer agents in COVID-19 will require more time.

## Author Contributions

VB was involved in the literature search, and drafting and preparation of the manuscript. BP was involved in the idea generation, and writing, checking, and approving of the manuscript. Both authors contributed to the article and approved the submitted version.

## Conflict of Interest

The authors declare that the research was conducted in the absence of any commercial or financial relationships that could be construed as a potential conflict of interest.
